# Our strategy to achieve and document reproducible computing

**DOI:** 10.1186/1471-2105-14-S17-A19

**Published:** 2013-10-22

**Authors:** Nisrine Enyinda, Zhifa Liu, Areg Negatu, Stan Pounds

**Affiliations:** 1 Department of Biostatistics, St. Jude Children’s Research Hospital, Memphis, TN 38105, USA; 2 Department of Computer Science, University of Memphis, Memphis, TN 38152, USA

## Background

The scientific and ethical importance of reproducible computing in analysis and interpretation of biomedical research data is now widely recognized. However, achieving and documenting reproducible computing is very challenging in a perpetually evolving research environment in which multiple users perform analyses of multiple data files on multiple platforms.

## Materials and methods

Here, we describe our three-component strategy to achieve and document permanent reproducible computing in our research environment. First, we use the Sweave literate programming infrastructure to embed R code and report text in the same file. Sweave performs the specified calculations in R, inserts those results directly into a LaTeX typesetting command file, and finally compiles the LaTeX typesetting file into a PDF file. Thus, a Sweave file internally documents the top-level R code that produces the reported results. However, a Sweave report does not retain its reproducibility if the input data files and lower-level R code are modified later. Therefore, as the second component of our strategy, we developed the Igloo system to archive and freeze files for permanent reproducibility. The Igloo system requests that the user document every file that is transferred to a frozen archive. The Igloo system freezes the files in an archive with a directory structure that annotates the files by research team (leukemia, brain tumor, etc) and category (code file, type of data file, etc). The archive directory is visible in our Windows and Linux high-performance computing environments and has permission controls to ensure appropriate access to the files. However, neither Sweave nor Igloo assists with the cumbersome task of identifying specific input files that should be frozen to ensure permanent reproducibility. As the third component of our strategy, we developed the R package rctrack that computationally tracks the accession and generation of files by an R analysis program. The rctrack package defines a function that identifies files which need to be frozen in order to ensure permanent reproducibility. Additionally, rctrack provides mechanisms to track and document the usage of other software for some calculations. Finally, the rctrack package defines a function that generates a Sweave appendix with details regarding the input data and code files and their impact on the reproducibility of the report.

## Results

By using and further enhancing these tools, we expect to achieve and document permanent and complete reproducibility of all our analyses in the very near future.

